# Mesenchymal Tumors Involving the Pancreas: A Clinicopathologic Analysis and Review of the Literature

**DOI:** 10.5146/tjpath.2022.01567

**Published:** 2022-01-21

**Authors:** Gokce Askan, Olca Basturk

**Affiliations:** Department of Pathology, Memorial Sloan Kettering Cancer Center, New York, USA

**Keywords:** Pancreas, Mesenchymal tumors, Benign, Malignant

## Abstract

*
Objective:* Most pancreatic tumors are epithelial, and, among these, more than 90% are of ductal origin. However, a variety of mesenchymal tumors may involve the pancreas and may manifest different clinicopathological characteristics. The literature on mesenchymal tumors in the pancreas is largely limited to individual case reports or analyses of small series, predominantly focusing on radiologic features.

*
Material and Method:* Authors’ institutional and consultation databases were reviewed to identify the mesenchymal tumors involving the pancreas.

*
Results:* Forty cases were identified; twenty-five (63%) tumors were benign/borderline, and the remaining fifteen (37%) were malignant. Of the benign/borderline tumors; 9 were solitary fibrous tumors, 6 gastrointestinal stromal tumors (GISTs), 4 schwannomas, 2 desmoid type fibromatosis, 1 lymphangioma, 1 ganglioneuroma, 1 inflammatory myofibroblastic tumor, and 1 low grade mesenchymal neoplasm. Malignant tumors included 6 cases of leiomyosarcomas, 4 liposarcomas, 2 rhabdomyosarcomas, 1 epithelioid angiosarcoma, 1 malignant peripheral nerve sheet tumor, and 1 undifferentiated pleomorphic sarcoma. Four cases (multicystic schwannoma, desmoid fibromatosis, lymphangioma and inflammatory myofibroblastic tumor) were preoperatively misdiagnosed as a primary epithelial tumor of the pancreas.

*
Conclusion:* Mesenchymal tumors rarely involve the pancreas. They are usually benign/borderline neoplasms but may be diagnostically challenging, especially clinically/radiologically, as they may form cystic and/or large lesions in the pancreas. Mesenchymal tumors should be considered in both the clinical/radiological and pathological differential diagnosis of pancreatic lesions.

## INTRODUCTION

The majority of the tumors involving the pancreas are of epithelial origin, and of these, pancreatic ductal adenocarcinomas (PDACs) are the most common primary tumors (1). However, mesenchymal tumors could involve the pancreas, too. Although imaging studies might be helpful to distinguish, some mesenchymal tumors might mimic epithelial ones very well (2). Moreover, radiologic findings could be misleading when the tumors present as a cystic and/or large lesion, as it gets difficult to identify the site of origin (1,3–5).

Primary mesenchymal tumors of the pancreas comprise only 1-2% of all pancreatic neoplasms (1), and the literature on mesenchymal tumors involving the pancreas is largely limited to case reports or analyses of small series (3,6–15). Here, we present a large series of mesenchymal tumors involving the pancreas, discuss their clinicopathologic features and differential diagnoses, and compare our findings with the previous experience reported in the literature.

## MATERIALS and METHODS

Pathology reports of autopsies and surgical pancreatic specimens from the authors’ institutional and consultation databases (1997-2020) have been reviewed to identify the mesenchymal tumors involving the pancreas.

Available gross photographs and descriptions as well as all histologic sections and immunohistochemical staining slides were re-evaluated to confirm the diagnosis. Available medical records, including imaging study reports, were reviewed to obtain clinical data including age, sex, presenting symptoms, treatment, and outcome. For the consultation cases, contributing physicians were contacted. Tumors that are metastatic to the pancreas from a remote site as well as tumors that were confined to the peripancreatic soft tissue or lymph nodes, without pancreatic involvement, were excluded.

### Statistical Analysis

Mean, median and ranges were used to describe quantitative variables. The Mann-Whitney U test or Fisher`s exact test was used to evaluate the differences in clinicopathologic features between benign/borderline and malignant tumors of the pancreas. *P*-values of <0.05 were considered statistically significant.

## RESULTS

### Cases

A total of forty cases were identified. Clinicopathologic features of these cases were summarized in Table 1.

**Table 1:** Clinicopathologic features of benign/borderline and malignant mesencyhmal tumors involving the pancreas.

	**Benign/borderline (n=25)**	**Malignant (n=15)**	**p value**
Mean age (year, range)	55 (19-80)	56 (2-74)	0.68
Female/Male	13/12	7/8	0.74
Tumor size (cm, range)	5 (0.5-16)	11 (4-20)	0.001
Pancreatic part involved Head Body/tail	17 4	4 9	0.01
Mean follow-up (months)	39	28	0.33
Status No evidence of disease (%) Alive with disease (%) Died of disease (%) Died of other causes (%)	18/20 (90) 1/20 (5) 0 (0) 1/20 (5)	4/8 (50) 2/8 (25) 2/8 (25) 0 (0)	

The mean age of the patients was 55 years for the entire cohort, younger than that of PDAC (mean age: 64 years). Twenty (50%) patients were female and twenty (50%) were male. Mean tumor size was 6 cm (range, 0.5-20 cm).

Twenty-five (63%) cases were classified as benign/borderline tumors, including nine solitary fibrous tumors (detailed analysis of these tumors is subject to another study) (16), six gastrointestinal stromal tumors (GISTs), four schwannomas (two (multi)cystic, two solid), two desmoid type fibromatosis, one lymphangioma, one ganglioneuroma, one inflammatory myofibroblastic tumor, and one low grade mesenchymal neoplasm. The remaining fifteen (37%) cases were malignant tumors, including six cases of leiomyosarcomas, four liposarcomas (three dedifferentiated liposarcomas, one pleomorphic liposarcoma), two rhabdomyosarcomas (one alveolar, one embryonal), one epithelioid angiosarcoma, one malignant peripheral nerve sheet tumor, and one undifferentiated pleomorphic sarcoma.

When benign/borderline and malignant mesenchymal tumors were compared, no gender predominance was identified in either group, and there was no statistically significant difference in the mean age of the patients (55 years vs. 56 years, respectively; p=0.68). The mean tumor size of benign/borderline mesenchymal tumors was smaller than that of malignant tumors (5 cm vs. 11 cm, respectively; p=0.001).

#### Clinical Findings

Detailed clinical information was available for twenty-five (63%) patients. The most common presenting symptoms were abdominal pain, loss of appetite, and weight loss. Four (16%) cases (solitary fibrous tumor, GIST, desmoid type fibromatosis, and dedifferentiated liposarcoma) were diagnosed incidentally during work-up for other intraabdominal pathologies. Four tumors (multicystic schwannoma, desmoid type fibromatosis, lymphangioma, and inflammatory myofibroblastic tumor) were clinically misdiagnosed (as mucinous cystic neoplasm, serous cystadenoma, lymphoepithelial cyst, and PDAC, respectively).

Two patients, one with GIST and another one with embryonal rhabdomyosarcoma, received neoadjuvant chemotherapy; one patient with leiomyosarcoma received neoadjuvant radiotherapy. Twenty patients underwent a pylorus-preserving pancreaticoduodenectomy: nine had a distal pancreatectomy, and ten had a local excision. One patient with leiomyosarcoma underwent autopsy.

#### Pathologic Findings

**Figure 1:**
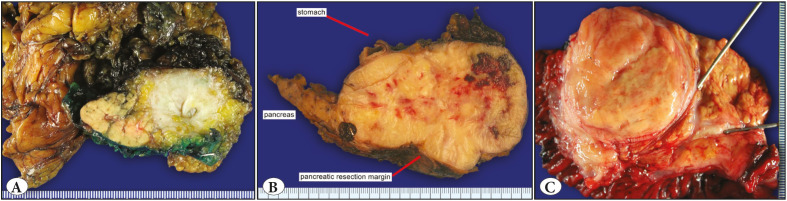
**A)** Desmoid type fibromatosis, characterized by a solid, ill-defined mass with tan-white cut surface, involving tail of the pancreas. **B)** Leiomyosarcoma involving the pancreas, peripancreatic adipose tissue, and the gastric wall. The mass is vaguely nodular and reveals hemorrhagic foci. **C)** Dedifferentiated liposarcoma involving head of the pancreas. The tumor is relatively well-circumscribed and has a fleshy cut surface.

Of the known thirty-six cases, twenty-one (58%) tumors were involving the head of the pancreas (Figure 1).

**Figure 2:**
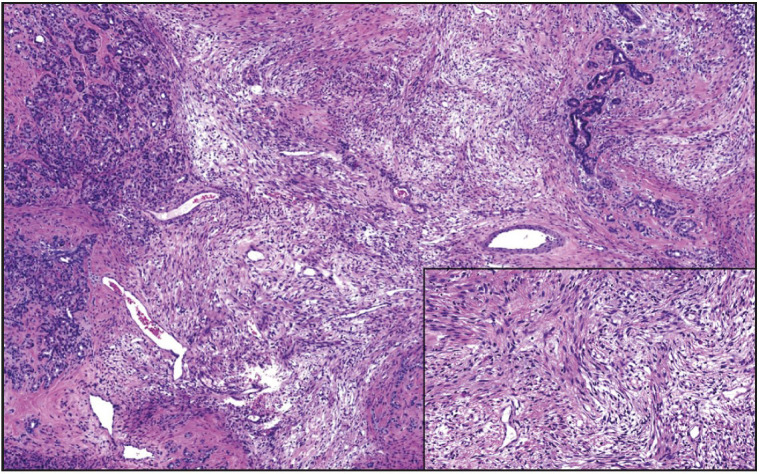
Solitary fibrous tumor composed of alternating zones of hypo and hypercellular areas and variably collagenous stroma (x100). Ovoid to fusiform spindled tumor cells, with indistinct cell borders and bland nuclei, are haphazardly distributed around dilated vascular structures (inset, x200).

**Figure 3:**
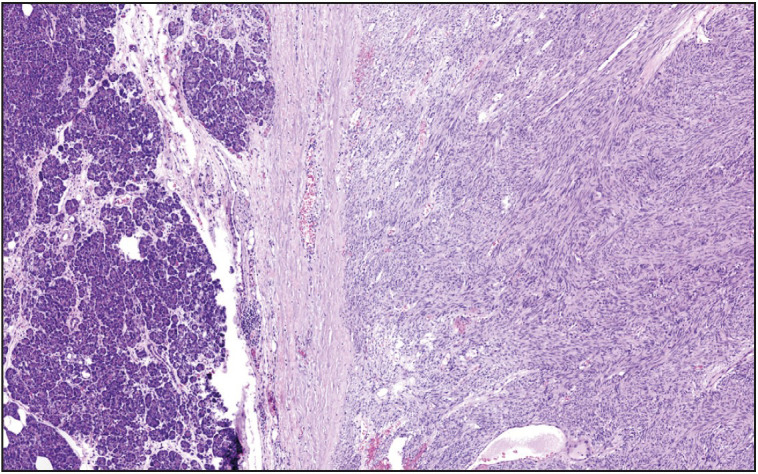
Gastrointestinal stromal tumor composed of spindle cells, with oval shaped nuclei and lightly eosinophilic cytoplasm, arranged in fascicles (x100).

**Figure 4:**
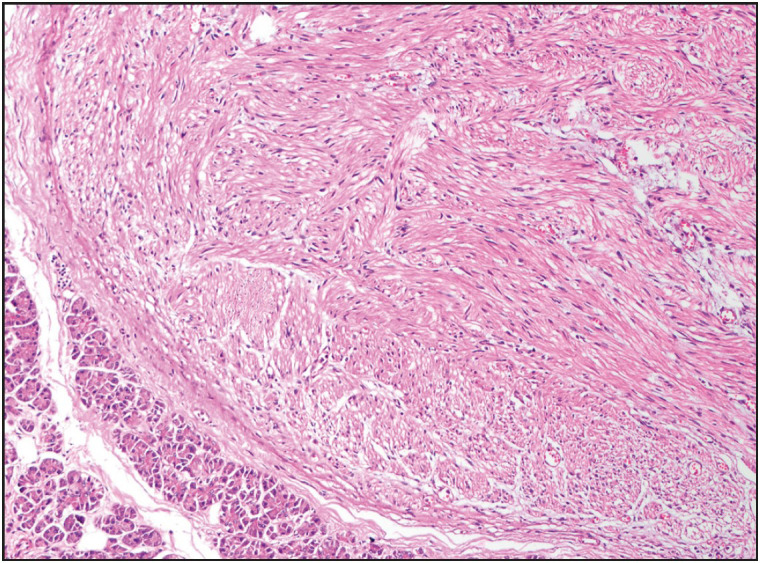
Schwannoma composed of interlacing bundles of spindle cells and collagen. The tumor cells have ill-defined, dense eosinophilic cytoplasm and ovoid to spindled nuclei (x200).

**Figure 5:**
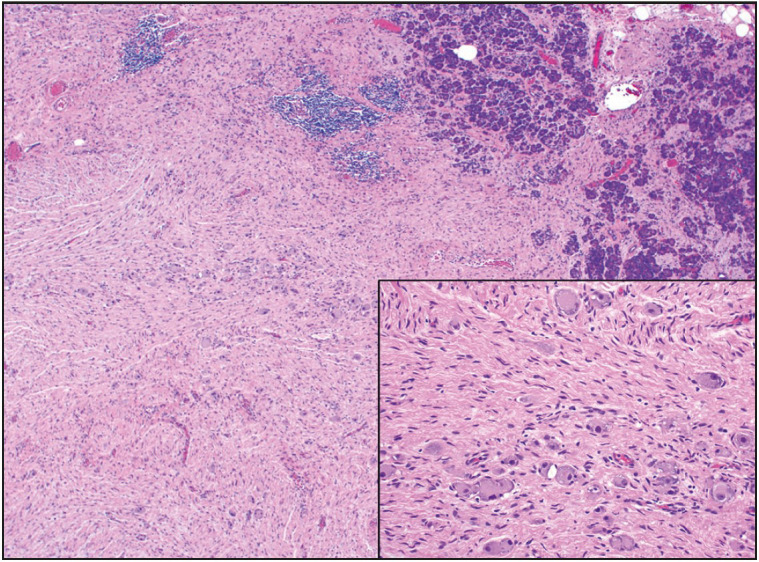
Ganglioneuroma composed of an admixture of schwann cells, with eosinophilic cytoplasm and wavy nuclei, arranged in a fascicular or whorled pattern (x100) and mature ganglion cells (inset, x200).

**Figure 6:**
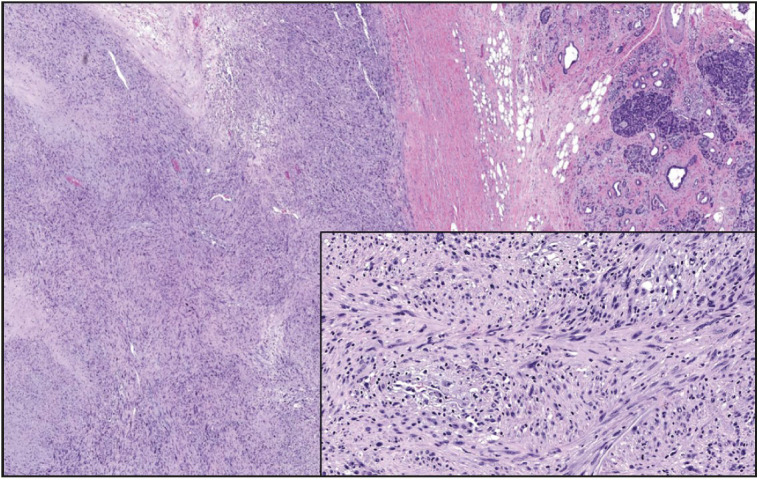
Leiomyosarcoma composed of palisading of tumor cells with prominent pleomorphism (x100). Tumor cells have oval to cigar-shaped, blunt-ended nuclei and light eosinophilic cytoplasm (inset, x200).

**Figure 7:**
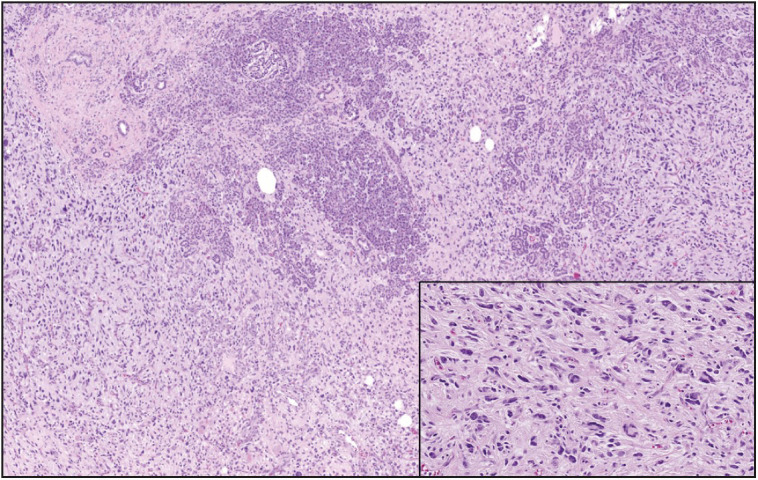
Pleomorphic liposarcoma involving pancreatic parenchyma (x100). Tumor cells have hyperchromatic bizarre nuclei (some in floret-like multinucleated giant cell forms) and light eosinophilic cytoplasm (inset, x200).

**Figure 8:**
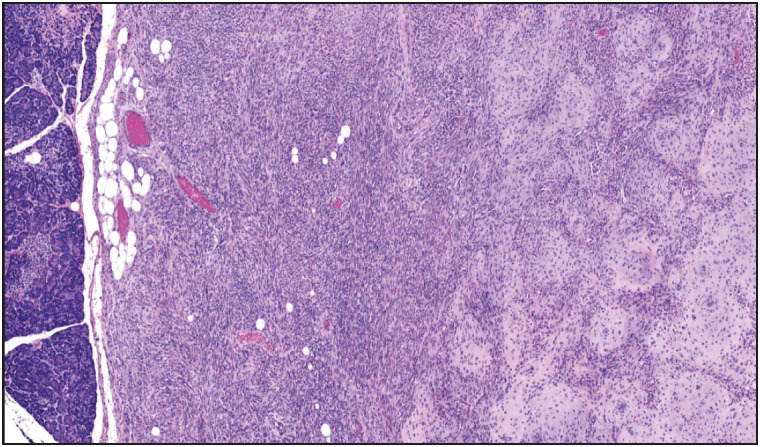
Malignant peripheral nerve sheet tumor composed of predominantly monomorphic spindle cells with scant to moderate eosinophilic, ill-defined cytoplasm arranged in fascicular and whorling pattern. Our case revealed heterologous chondromatous differentiation (x100).

Pathologic features of the majority of the tumors were identical to those of mesenchymal tumors arising from soft tissue or other organs (Figure 1 Figure 2 Figure 3 Figure 4 Figure 5 Figure 6 Figure 7 Figure 8). Solitary fibrous tumors (Figure 2) characteristically revealed variable labeling with CD34 and STAT6 immunohistochemical stains. GISTs (Figure 3) showed positivity for CD117, DOG1, SMA, and CD34. Moreover, two GISTs revealed *KIT* and another one revealed *PDGFRA *mutations. Schwannomas (Figure 4) demonstrated S100 protein expression. Fibromatoses were confirmed by nuclear beta-catenin staining. Lymphangioma labeled with CD31. Schwann cells and ganglion cells of ganglioneuroma (Figure 5) were positive for S100 and neurofilament proteins, and synaptophysin. The inflammatory myofibroblastic tumor revealed ALK expression. Leiomyosarcomas (Figure 6) showed diffuse and strong immunoreactivity for SMA and desmin. Liposarcomas (Figure 7) were positive for CDK4 and MDM2 immunohistochemical stains, and the only case tested was found to harbor *CDK4 *and* MDM2* mutations. Rhabdomyosarcomas expressed vimentin, desmin, myoD1, and myogenin. Epithelioid angiosarcoma was diffusely and strongly positive for vascular markers, CD31, Factor VIII, and Fli-1, as well as for keratin. Of note, this case also had a minute solid pseudopapillary neoplasm (SPN) of the pancreas; however, the tumors were morphologically distinct, and the immunohistochemical staining of the SPN component was quite characteristic, with vimentin, alpha 1 antitrypsin, CD10, and nuclear beta-catenin expression. Moreover, the SPN component was negative for vascular markers and keratin.

Another case revealed a predominantly monomorphic spindle cell neoplasm arranged in intersecting long fascicles, associated with areas of necrosis and high mitotic activity. A focus of cartilaginous divergent differentiation was also identified (Figure 8). By immunohistochemistry, the tumor cells were positive for S100 protein, while negative for desmin, myogenin, SOX10, MDM2, and CDK4. *KIT *and *PDGFRA* mutation analyses were also negative. FISH studies, performed to rule out the possibility of gastrointestinal clear cell sarcoma and a myoepithelial tumor, showed no rearrangement of the *EWSR1, FUS, ATF1, and CREB1 *genes. Based on the morphology and immunoprofile, the tumor was classified as a high grade malignant peripheral nerve sheath tumor with divergent chondrosarcomatous differentiation.

**Figure 9:**
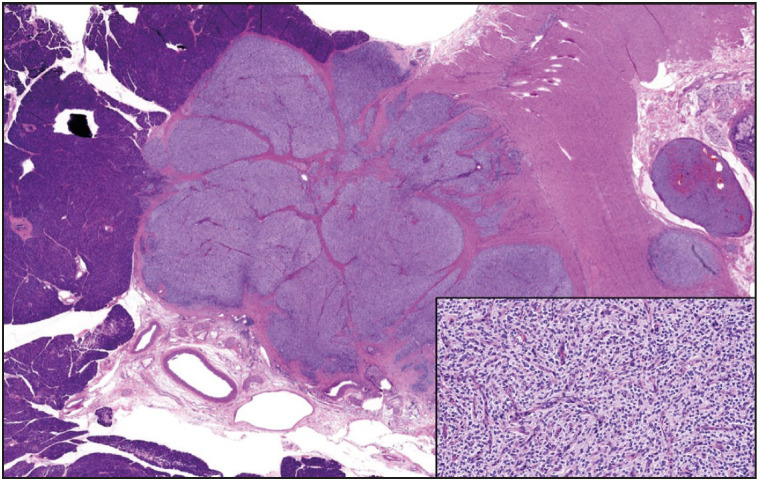
Low grade mesenchymal neoplasm involving pancreatic parenchyma and duodenal wall (x100). The tumor is composed of monomorphic cells with ovoid to round nuclei, arranged in a vaguely nested pattern. Increased vascularity is also noted (inset, x200). This tumor could not be further characterized despite extensive work-up.

There were two unusual cases: one multinodular tumor was composed of monomorphic cells with ovoid to round nuclei, arranged in a vaguely nested pattern (Figure 9). Despite extensive work-up the tumor could not be further characterized and classified as a low-grade mesenchymal neoplasm. (Immunohistochemical stains showed that the tumor cells were positive for desmin and TFE3, while negative for pancytokeratin, CAM5.2, EMA, chromogranin, synaptophysin, NSE, CD45, SMA, HHF35, myogenin, CD117, DOG1, MUC4, S100, SOX10, HMB45, Melan-A, CD31, ERG, TLE1, STAT6, and Cathepsin-K). Beta-catenin revealed membranous staining. The Ki67 proliferative index was less than 5%. FISH studies showed no rearrangement of the *EWSR1, FUS, GLI1, TFE3, NOTCH2, NCOA2, *and* PHF* genes. Targeted next generation sequencing involving all targeted (≥ 400) genes did not reveal any somatic mutations or amplifications/homozygous deletions in any known oncogenes or tumor suppressor genes. The Archer FusionPlex assay did not detect any recurrent likely pathogenic gene fusions). Another tumor was composed of pleomorphic spindle cells with malignant features including necrosis, high mitotic counts, and nuclear atypia. The tumor did not reveal any specific differentiation (immunohistochemical stains showed that the tumor cells were negative for pancytokeratin, SMA, desmin, CD117, DOG1, CD34S100, Melan-A, and HMB45). FISH analysis for the detection of *MDM2* gene amplifications was also performed to rule out the possibility of dedifferentiated liposarcoma, and the result was negative. Based on these, the tumor was classified as an undifferentiated pleomorphic sarcoma.

Moreover, two benign (desmoid type fibromatosis and ganglioneuroma) and four malignant (three dedifferentiated liposarcomas and one embryonal rhabdomyosarcoma) tumors had positive surgical margin(s). Five tumors (two rhabdomyosarcomas, two GISTs, and one solitary fibrous tumor) were found to have metastatic lymph node(s). The tumor diameter for GISTs with metastatic lymph node was 4 cm and 5 cm, and the mitotic count was 1 mitosis per 50 high power fields and 3 mitoses per 50 high power fields, respectively. Of note, the case with the largest tumor (16 cm) had no lymph node metastasis.

Moreover, one GIST and the epithelioid angiosarcoma had concurrent tumors in other organs: an ampullary adenocarcinoma and an appendiceal neuroendocrine tumor, respectively.

### Outcome

Clinical follow-up was available for twenty-eight (70%) cases. The mean follow-up was 37 months for the entire cohort (range, 3-140 months).

Follow-up information was available for twenty patients with a benign/borderline mesenchymal tumor. Eighteen (90%) patients are alive with no evidence of disease, with a mean follow-up 39 months. One patient with a GIST had distant metastasis after 68 months and is alive. The remaining one died of other causes.

Follow-up information was available for eight patients with a malignant mesenchymal tumor. Four (50%) patients are alive with no evidence of disease, with a mean follow-up 28 months. Two (25%) patients with dedifferentiated liposarcoma had a local recurrence after 6 and 48 months, respectively and are alive. Two (25%) patients, one with a leiomyosarcoma and one with a malignant peripheral nerve sheet tumor, had distant metastasis after 4 months and both patients died of disease after 8 months.

## DISCUSSION

Mesenchymal tumors rarely involve the pancreas and most of them are believed to be secondary lesions (1,17). Our experience is mainly based on individual case reports, analyses of small series of cases, or opinions presented in textbooks, predominantly focusing on radiologic features (3,6–14,17,18). To the best of our knowledge, our study is the largest study documenting clinicopathologic features of mesenchymal tumors involving the pancreas.

In the literature, the most commonly reported primary benign/borderline mesenchymal tumors were schwannoma followed by inflammatory myofibroblastic tumor, whereas the most commonly reported malignant ones were leiomyosarcoma and undifferentiated sarcomas (3,6–11,19–23). In our series, the most common benign/borderline mesenchymal tumors involving the pancreas were solitary fibrous tumor (n=9), followed by schwannoma (n=4), and the most common malignant ones were leiomyosarcoma (n=6), followed by liposarcoma (n=4).

Mesenchymal tumors can mimic epithelial tumors of the pancreas and the patients’ demographics, clinical symptoms, or tumor location are not helpful to distinguish these two tumor categories. In our series, the mean age was 55 years, younger than that of PDAC, but the most common presenting symptom was abdominal pain in both groups. Moreover, radiologically, mesenchymal tumors may closely mimic pancreatic epithelial tumors, making the preoperative diagnosis difficult (3–5,17,24). Therefore, surgical resection and histologic examination is necessary. For example, desmoid type fibromatosis is a benign but locally agressive tumor, mostly located in the pancreatic tail (25–28). Radiologically, it can mimic PDAC due to obstruction of the pancreatic duct (28–30). Interestingly, one of the desmoid type fibromatosis cases in our series was preoperatively diagnosed as microcystic serous cystadenoma. Fortunately, these tumors are histologically very different.

When they are cystic, mesenchymal tumors may mimic pancreatic epithelial tumors not only radiologically but also histologically. Schwannomas and lymphangiomas are the most common mesenchymal tumors that can present as cystic lesions and mimic the cystic epithelial tumors such as intraductal papillary mucinous neoplasm, mucinous cystic neoplasm, serous cystadenoma, and cystic neuroendocrine tumor (15,31). One of our schwannoma cases presented as a multicystic lesion and not only clinically but also histologically mimicked mucinous cystic neoplasm (spindle cells mimicked ovarian-type stroma). Immunohistochemical stains were helpful (the tumor cells were positive for S100, while negative for SMA, desmin, ER, and PR) to exclude the possibility of mucinous cystic neoplasm. The lymphangioma case that presented as a cystic lesion in our series was also clinically misdiagnosed as a lymphoepithelial cyst (31–33). Morphology and immunohistochemical stains were helpful to confirm the lymphatic nature of the tumor (the tumor cells were positive for CD31, CD34, and factor VIII, while negative for PanCK).

Malignant mesenchymal tumors could be also very challenging because the possibility of sarcomatoid carcinoma must be excluded by extensive, if not total, sampling, and careful microscopic examination is required to search for epithelial components, which might be very focal. Immunohistochemical stains and molecular studies are also frequently necessary, especially if the malignant tumors in the differential diagnosis have a specific immunoprofile or molecular features. For example, presence of MDM2 protein expression and/or *MDM2* gene amplifications confirm the diagnosis of dedifferentiated liposarcoma (2,3,34–36). In our series, there were three liposarcomas; two were dedifferentiated and one was of the pleomorphic subtype. Patients with dedifferentiated liposarcoma had positive surgical margin(s) and developed a local recurrence, after 10 months and 53 months, respectively.

Interestingly, in our series, there were also two cases of rhabdomyosarcoma, one embryonal and one alveolar subtype, which were consultation cases. The ages of the patients were 2 and 19 years, respectively and both tumors were located in the pancreatic head. Rhabdomyosarcomas are malignant tumors arising from the embryonic mesenchyme with the potential to differentiate into skeletal muscle. They are most commonly seen in infants and children. The pancreas is a very unusual site for this tumor, and only a few cases have been described in the literature (37). This rare entity should be kept in mind for children and young adults with an abdominal mass to expedite the diagnosis and start the additional treatment as they are chemosensitive tumors and surgical treatment is followed by chemoradiation therapy (38,39). Follow-up information was available for only one of our patients with rhabdomyosarcoma, who received chemoradiation therapy and the patient has no evidence of disease after 4 months.

In conclusion, mesenchymal tumors rarely involve the pancreas. They could present as a solid or a cystic mass and preoperative diagnosis is usually challenging as their radiologic findings may not be specific. These tumors may mimic the pancreatic epithelial neoplasms even histologically. In our series, four patients, all with a benign/ borderline mesenchymal tumor, were clinically misdiagnosed. Histopathological examination and extensive ancillary studies are necessary for a definite diagnosis.

## Conflict of Interest

The authors declare no conflict of interest.
